# Quantitative analysis of the 3-d morphology of adolescent idiopathic scoliosis with implications for surgical strategy

**DOI:** 10.1186/1748-7161-10-S1-O65

**Published:** 2015-01-19

**Authors:** Tom P Schlösser, Marijn Van Stralen, Winnie W Chu, Tsz-Ping Lam, Bobby K Ng, Koen L Vincken, Jack C Cheng, René M Castelein

**Affiliations:** 1 Department of Orthopaedic Surgery, University Medical Center Utrecht, Utrecht, the Netherlands; 2 Image Sciences Institute, University Medical Center Utrecht, Utrecht, the Netherlands; 3 Department of Diagnostic Radiology and Organ Imaging, Prince of Wales Hospital, The Chinese University of Hong Kong, Shatin, Hong Kong; 4 Department of Orthopaedics and Traumatology, Prince of Wales Hospital, The Chinese University of Hong Kong, Shatin, Hong Kong

## Objective

AIS is a three-dimensional (3-D) spinal deformity, characterized by lateral deviation, axial rotation and apical lordosis of three individual curves. The 3-D morphology of the different areas of the scoliotic spine has not been quantified so far. This is the first study to accurately define the 3-D morphology of AIS and compare it to normal anatomy. This is important for understanding the true nature of the problem and for harmonious 3-D surgical correction to avoid complications such as thoracic hypokyphosis and junctional kyphosis.

## Material and methods

A unique series of high-resolution CT scans of 77 AIS patients and 22 matched controls was used for this study. Non-idiopathic curves were excluded. Scans were obtained in prone position for navigation purposes. True transverse sections were reconstructed taking rotation, and coronal and sagittal tilt into account. Using semi-automatic analysis software, 'endplate-vectors' were calculated and complete 3-D spine reconstructions were acquired. Coronal deviation, axial rotation and the exact anterior-posterior length discrepancy, as defined per vertebra and disc in 3-D, were measured for each curvature and for the junctional segments, semi-automatically. Intraclass correlation coefficients for intraobserver reliability were 0.98-1.00.

## Results

All thoracic and (thoraco) lumbar curves were longer anteriorly (+3.8% and +9.4%, respectively), while the proximal and distal junctional segments between the scoliotic curves were straight, with a tendency to slight kyphosis in the proximal junctional segment. The same thoracic segments in the controls were shorter anteriorly (-4.1%; P<0.001). Linear relations were observed between the upright radiographic Cobb angles, axial rotation and the anterior-posterior length discrepancy on the CT scans in thoracic curves (r>0.729; P<0.001) and (thoraco) lumbar curves (r>0.485; P<0.001). Lateral radiographs had no value for prediction of the 3-D morphology of AIS.

## Conclusion

AIS consists of three separate, rotated, lordotic curves and two straight junctional segments. Anterior overgrowth is regional rather than global, and the disc contributes more than the vertebral body. This study provides guidelines for the amount of posterior lengthening and/or anterior shortening that is necessary for true harmonious 3-D realignment.

